# Glycine Receptor Subtypes and Their Roles in Nociception and Chronic Pain

**DOI:** 10.3389/fnmol.2022.848642

**Published:** 2022-03-23

**Authors:** Victoria P. San Martín, Anggelo Sazo, Elías Utreras, Gustavo Moraga-Cid, Gonzalo E. Yévenes

**Affiliations:** ^1^Department of Physiology, Faculty of Biological Sciences, University of Concepcion, Concepcion, Chile; ^2^Millennium Nucleus for the Study of Pain (MiNuSPain), Santiago, Chile; ^3^Department of Biology, Faculty of Science, Universidad de Chile, Santiago, Chile

**Keywords:** glycine receptor (GlyR), chronic pain, nociception, phosphorylation, synaptic plasticity

## Abstract

Disruption of the inhibitory control provided by the glycinergic system is one of the major mechanisms underlying chronic pain. In line with this concept, recent studies have provided robust proof that pharmacological intervention of glycine receptors (GlyRs) restores the inhibitory function and exerts anti-nociceptive effects on preclinical models of chronic pain. A targeted regulation of the glycinergic system requires the identification of the GlyR subtypes involved in chronic pain states. Nevertheless, the roles of individual GlyR subunits in nociception and in chronic pain are yet not well defined. This review aims to provide a systematic outline on the contribution of GlyR subtypes in chronic pain mechanisms, with a particular focus on molecular pathways of spinal glycinergic dis-inhibition mediated by post-translational modifications at the receptor level. The current experimental evidence has shown that phosphorylation of synaptic α1β and α3β GlyRs are involved in processes of spinal glycinergic dis-inhibition triggered by chronic inflammatory pain. On the other hand, the participation of α2-containing GlyRs and of β subunits in pain signaling have been less studied and remain undefined. Although many questions in the field are still unresolved, future progress in GlyR research may soon open new exciting avenues into understanding and controlling chronic pain.

## The Glycinergic System

Glycine receptors (GlyRs) are pentameric ligand-gated ion channels (pLGICs). The binding of the neurotransmitter glycine to the receptor protein opens a chloride-permeable pore, allowing the hyperpolarization of the membrane potential (Lynch, 2009; Zeilhofer et al., 2012). Functional GlyRs can be composed exclusively by α subunits (i.e., homomeric) or by α and β subunits (i.e., heteromeric). Four isoforms of the a subunits (α1-4) and one β subunit have been identified (Lynch, 2009; Zeilhofer et al., 2012, 2018). The a subunits display a high level of amino acid sequence identity (>80% by comparing human α1, α2, and α3 subunits) and share the prototypical structure of a pLGIC subunit: an amino-terminal extracellular domain (ECD), four transmembrane (TM1-4) domains, and a large intracellular domain between the TM3 and TM4 domains (ICD). Each subunit contains an ECD and an ICD that control the agonist binding and intracellular modulation, while the TM2 domains shape the ion pore (Du et al., 2015; Huang et al., 2015). The β subunit does not form functional homomeric channels but is key for GlyR clustering and stabilization at synapses through the interaction of the ICD of β subunit with the scaffolding protein gephyrin (Tyagarajan and Fritschy, 2014; Groeneweg et al., 2018). Growing progress in structural biology have allowed the resolution of diverse GlyR subtypes structures, including homomeric and heteromeric GlyRs (Du et al., 2015; Huang et al., 2015; Yu et al., 2021; Zhu and Gouaux, 2021). In parallel, GlyR pharmacology has been also expanded in recent years (Burgos et al., 2016; Cioffi, 2017). Interestingly, the combination of structural biology with receptor ligands have yielded structures showing the binding sites for agonists, antagonists, and a synthetic modulator, and thus providing mechanistic insights into GlyR function (Huang et al., 2015, 2017; Zhu and Gouaux, 2021).

Other key proteins of the glycinergic system are the plasma membrane glycine transporters (GlyT1 and GlyT2) and the vesicular amino acid transporter VGAT/VIAAA (Zeilhofer et al., 2012; Marques et al., 2020). GlyT2 is abundantly expressed in the spinal cord and brain stem. This distribution profile matches well with the presence of prominent phasic glycinergic activity, which is translated into detectable glycinergic inhibitory post-synaptic currents (Gly-IPSCs). The existence of Gly-IPSCs is also supported by the widespread expression of α1 and β GlyR subunits along the spinal cord (Zeilhofer et al., 2012). Interestingly, the distribution of α3-containting GlyRs is restricted to the superficial laminae of dorsal horn, where they are possibly integrated in mixed post-synaptic domains together with α1 and β subunits (Harvey et al., 2004). Apart from fast synaptic inhibition, it has been reported that GlyRs modulate neuronal excitability via tonic inhibition (McCracken et al., 2017; Molchanova et al., 2018; Muñoz et al., 2020) and presynaptic modulation (Turecek and Trussell, 2001; Jeong et al., 2003).

Glycine receptors activation controls key neurophysiological functions, such as respiratory rhythm, muscle tone and motor coordination (Lynch, 2009; Callister and Graham, 2010; Zeilhofer et al., 2012). Cumulative data from human studies and mouse models have helped to establish the neurophysiological relevance of GlyR subunits in health and disease. A well stablished example of the pathological GlyR relevance is human hyperekplexia, a neuromotor disorder frequently associated with genetic alterations in the genes encoding α1 and β GlyRs (*Glra1* and *Glrb*) (Bode and Lynch, 2014). Other studies have shed light on the physiological roles of GlyRs composed of α2 and α3 subunits. These studies have determined that α2 GlyRs are involved in cortical migration, neurogenesis, and recognition memory (Avila et al., 2013; Pilorge et al., 2016; Lin et al., 2017), while GlyRs containing α3 subunits contribute to breathing control and hearing (Manzke et al., 2010; Dlugaiczyk et al., 2016; Tziridis et al., 2017). Noteworthy, additional reports have revealed central roles of α2 and α3 GlyRs in neurological diseases, including autism (Pilorge et al., 2016), epilepsy (Winkelmann et al., 2014) and alcohol addiction (Gallegos et al., 2021).

## Glycinergic Neurotransmission in Chronic Pain

Chronic pain is a widespread pathological state that affects around 20% of the adult population worldwide. Different peripheral and central mechanisms contribute to chronic pain (Zeilhofer et al., 2012; Yekkirala et al., 2017). The role of the glycinergic system in pain has been extensively studied in the dorsal horn. Pioneering studies using intrathecal injections of strychnine were the first to suggest a role of spinal GlyRs in pain control (Beyer et al., 1985; Yaksh, 1989). The GlyR activity on dorsal horn is also critical to set a correct maturation of the neural circuits controlling pain and touch (Koch et al., 2012; Koch and Fitzgerald, 2013). Additional studies have shown that the selective ablation and chemogenetic manipulation of dorsal horn glycinergic neurons exert a direct control of pain and itch (Foster et al., 2015). These results together with others (Miraucourt et al., 2007; Lu et al., 2013; Petitjean et al., 2015) have established the contribution of the glycinergic system to the spinal control of innocuous and painful signals.

Further insights have demonstrated that the glycinergic tone in the dorsal horn is diminished in chronic pain models. The glycinergic dis-inhibition has been shown in paradigms of persistent pain induced by peripheral inflammation, nerve injury and diabetes-induced neuropathy (Harvey et al., 2004; Chiu et al., 2016; Imlach et al., 2016; Zhang et al., 2019a). In line with these observations, the exogenous application of pain-related mediators (e.g., PGE2 and IL1β) to naïve spinal tissue also generated short-term alterations of glycinergic neurotransmission (Ahmadi et al., 2002; Kawasaki et al., 2008: Chirila et al., 2014).

The mechanisms underlying the glycinergic dysfunction in chronic pain are related to diverse plastic changes at peripheral and central sites. These processes include, for instance, apoptosis of inhibitory neurons, alterations of chloride homeostasis, microglial proliferation and activation, long-term potentiation or depression, activity-dependent C-fiber stimulation, and post-translational modification of inhibitory receptors (Scholz et al., 2005; Pernia-Andrade et al., 2009; Thacker et al., 2009; Kim et al., 2012; Zeilhofer et al., 2012; Chirila et al., 2014; Zhang et al., 2019a).

The evidence discussed above has led to the development of pharmacological tools for the restoration of glycinergic activity. Recent studies have provided robust proof-of-concept that positive allosteric modulators (PAMs) of GlyRs exert anti-nociceptive effects on preclinical models of chronic pain (Cioffi, 2017; Zeilhofer et al., 2018, 2021). Thus, it is likely that the integration of basic research and translational approaches with focus on the glycinergic system may identify novel drugs against chronic pain.

## Glycine Receptor Subtypes Involved in Nociception and Chronic Pain

**FIGURE 1:**
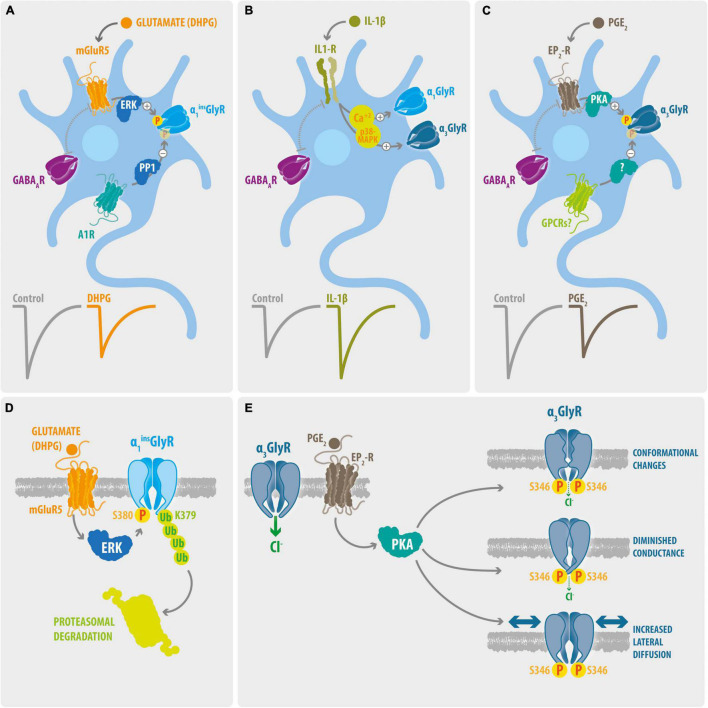
Signaling pathways and molecular mechanisms involved on processes of glycinergic disinhibition in chronic pain. **(A)** *Phosphorylation of dorsal horn GlyRs containing the α1*^ins^* subunit*. The activation of mGluR5 triggers the ERK-dependent phosphorylation of synaptic α1*^ins^* GlyRs. On the other hand, activation of the A1Rs promotes its PP1-mediated de-phosphorylation. Activation of mGluR5 signaling with DHPG decreases the amplitude of Gly-IPSCs, whereas GABA-IPSCs were not affected. **(B)** *IL-1β potentiation of glycinergic synapses.* The IL-1β mediated activation of IL1-R generates a long-term potentiation of the Gly-IPSCs on dorsal horn GABAergic neurons. The glycinergic potentiation involves increases on intracellular Ca^2+^ and p38-MAPK activity. The IL-1β signaling did not affect the GABA-IPSCs. Whether this mechanism preferentially target synaptic α1 or α3-containing GlyRs is unknown. **(C)** *Phosphorylation of dorsal horn GlyRs containing the α3 subunit*. Activation of EP2-R by PGE_2_ decreases the Gly-IPSCs on dorsal horn neurons through the PKA-mediated phosphorylation of synaptic α3 GlyRs. The stimulation of EP2-R did not modify the GABA-IPSCs. The molecular identity of potential GPCRs involved in the de-phosphorylation of dorsal horn α3GlyRs remains undetermined. **(D)** *Molecular mechanisms underlying the mGluR5-mediated glycinergic dis-inhibition*. Activation of mGluR5 stimulate the binding of ERK to a consensus site located on the splice cassette (^380^SPMLNLPQ) within the ICD of α1*^ins^*GlyR. ERK targets and phosphorylate the S380 residue and stimulate the subsequent ubiquitination of the L379 residue through the E3 ubiquitin ligase HUWE, triggering the proteasomal degradation of α1*^ins^* GlyRs. **(E)** *Molecular mechanisms underlying the EP2-R mediated glycinergic dis-inhibition*. Activation of EP2-Rs triggers the PKA-dependent phosphorylation of S346 of α3GlyRs, leading to alterations on the ion channel function and to enhanced lateral mobility of the receptor.

A targeted pharmacological intervention in the glycinergic system requires the identification of GlyR subtypes involved in chronic pain states of different origins. The absence of subunit selective GlyR pharmacological agents (Yevenes and Zeilhofer, 2011; Cioffi, 2017) and the limited quantity of genetically modified mouse models have hampered our insights into the contribution of specific GlyR subtypes in disease states. Despite these limitations, recent reports have shed light on GlyR subunits involved in mechanisms of spinal glycinergic dysfunction in chronic pain (summarized in Figure 1 and Table 1).

**TABLE 1:** Participation of GlyR α subunits in nociception and chronic pain states.

GlyR subunit	Species	Behavioral models	Genetic modifications	Main technical approaches	References
α1	Mouse	Complete Freund’s adjuvant model of inflammatory pain	Transgenic EGFP-expressing inhibitory neuron (GIN) line	Electrophysiology Immunocytochemistry	Takazawa et al., 2017
	Human	Not applicable	Diagnosed hyperekplexia (mutations *on Glra1* and *Grlb* genes)	Battery of quantitative sensory tests	Vuilleumier et al., 2018
	Mouse	Formalin model of inflammatory pain	None	Electrophysiology Immunocytochemistry Inmunoprecipitation Gene knock-down Behavioral tests	Zhang et al., 2019a
	Mouse	Complete Freund’s adjuvant model of inflammatory pain	None	Biotinylation Electrophysiology Gene knock-down	Diao et al., 2020

α2	Mouse	Formalin, Zymosan A and Complete Freund’s adjuvant models of inflammatory pain Spare nerve injury model of neuropathic pain	Global knock-out	*In situ* hybridization Behavioral tests	Kallenborn-Gerhardt et al., 2012
	Rat	Partial nerve ligation model of neuropathic pain	None	Electrophysiology Western blot	Imlach et al., 2016
	Mouse	Spinal nerve ligation model of neuropathic pain	None	RNA-Seq	Yu et al., 2019

α3	Mouse	Zymosan A and Complete Freund’s adjuvant models of inflammatory pain	Global knock-out	Electrophysiology Behavioral tests Immunocytochemistry	Harvey et al., 2004
	Mouse	Formalin model of inflammatory pain Chronic constriction injury model of neuropathic pain	Global knock-out	Behavioral tests	Hösl et al., 2006
	Mouse	Zymosan A model of inflammatory pain Chronic constriction injury model of neuropathic pain	α3GlyR-S346A knock-in	Electrophysiology Behavioral tests	Werynska et al., 2021

### Glycine Receptors Composed of α1 Subunit

As briefly described above, α1GlyRs are widely expressed along the spinal cord and brainstem and contribute to essential physiological functions. Accordingly, the global genetic inactivation of the *Glra1* gene is lethal, and mutations in *Glra1* cause hyperekplexic phenotypes in mice (Schaefer et al., 2013; Bode and Lynch, 2014). Nonetheless, a recent report has established the significance of α1GlyRs on pain processing in humans by studying patients with hyperekplexia (Vuilleumier et al., 2018). Using a battery of quantitative sensory tests, this study showed that patients with diverse mutations in the *Glra1* gene have lower pain thresholds than healthy volunteers.

A diminished glycinergic transmission in the superficial dorsal horn of inflamed rodents has been found in studies from different groups (Chirila et al., 2014; Takazawa et al., 2017; Zhang et al., 2019a). Although the prominent expression of synapses containing α1β GlyRs on the dorsal horn suggest an expected main role of α1-containing GlyRs in pain plasticity, experimental proof of spinal dis-inhibition processes directly driven by modifications on α1GlyRs were not reported until recently. A key article by Zhang et al. (2019a,b) demonstrated that ERK-mediated phosphorylation of the S380 residue within the longer splice variant of α1GlyR, α1*^ins^*GlyR, promotes the ubiquitination and endocytosis of the receptor, leading to a reduction of glycinergic neurotransmission (Figures 1A–D). A role of this pathway in inflammatory pain was determined using the formalin model. The dorsal horn ipsilateral to the formalin injection showed an enhanced phosphorylation and ubiquitination of α1*^ins^*GlyRs, and intrathecal application of a synthetic peptide comprising the putative ERK docking domain of α1*^ins^*GlyRs attenuated the second-phase nociceptive behaviors of formalin-injected mice (Zhang et al., 2019a). In contrast, other studies have shown that altered glycinergic neurotransmission does not necessarily correlate with a weakened expression of spinal GlyRs. For example, Takazawa et al. (2017) showed that glycinergic dis-inhibition triggered by peripheral inflammation takes place without detectable changes either in the density of inhibitory synapses or in the percentage of inhibitory synapses containing α1GlyR subunits, suggesting the absence of a significant receptor endocytosis.

Further studies have shown that the phosphorylation state of the S380 residue of α1*^ins^*GlyRs on the dorsal horn is dynamically controlled by G-protein coupled receptors (GPCRs) (Figure 1A). Bai et al. (2017) reported the potentiation of Gly-IPSCs mediated by the activation of adenosine A1 receptor (A1R). Using chemical inhibitors and recombinant GlyRs, the authors showed that A1R activation potentiated glycine-evoked currents of α1*^ins^*GlyR, but not of α1GlyRs. In a follow up study, the same research group showed that intrathecal application of adenosine reduced the phosphorylation of S380 of α1*^ins^*GlyRs in inflamed mice and attenuated pain hypersensitivity through a mechanism involving dephosphorylation of α1*^ins^*GlyRs by protein phosphatase-1 (PP1) (Diao et al., 2020). On the other hand, it was reported that the activation of mGluR5 reduces dorsal horn Gly-IPSCs by specifically promoting ERK mediated phosphorylation of S380 of α1*^ins^*GlyRs (Zhang et al., 2019a). Together, these studies suggest that the phosphorylation state of the S380 residue of α1*^ins^*GlyRs determines the strength of glycinergic neurotransmission in the dorsal horn.

Another signaling pathway modulating spinal glycinergic synapses was described by Chirila et al. (2014). The authors demonstrated the presence of a rapid potentiation of Gly-IPSCs on GABAergic neurons following the acute application of interleukin-1β (IL-1β) (Figure 1B). Interestingly, others have explored the mechanism underlying IL-1β induced glycinergic plasticity at the molecular level (Patrizio et al., 2017). Using tagged GlyR subunits combined with photoactivated localization microscopy (PALM) and single molecule tracking, Patrizio and collaborators determined that IL-1β reduced the number of α1-containing GlyRs at spinal synapses, while the synaptic occupancy of α3-containing GlyRs was not modified. Hence, although these data sets appear to be contradictory, the current evidence suggests that inflammatory mediators involved in pain processes may exert diverse plastic modifications on glycinergic synapses.

### Glycine Receptors Composed of α2 Subunit

The contribution of α2-containing GlyRs to nociception and chronic pain is not yet clear. Considering the very low or absent expression of α2GlyRs in the dorsal horn of adult mammals (Lynch, 2009; Zeilhofer et al., 2012), a major contribution of the α2 subunits to spinal pain processing seems unlikely. However, mice lacking the expression of α2GlyRs (α2^–/–^) have given first insights into its potential relevance. Behavioral assessments performed in α2^–/–^ mice showed an altered pain sensitization recovery after peripheral inflammation with Zymosan A (Kallenborn-Gerhardt et al., 2012). Further assays showed an unaltered pain hypersensitivity in other two models of inflammatory pain. The α2^–/–^ mice displayed normal acute nociception and unchanged pain sensitization after peripheral nerve injury. Other studies have shown that α2GlyRs may participate in the plasticity mechanisms underlying neuropathic pain. Imlach et al. (2016) showed that nerve injury induced an enhanced expression of α2GlyRs in a subset of dorsal horn neurons (identified as radial neurons), which correlated well with a reduced efficacy of glycinergic transmission. In line with these observations, a report based on RNA-Seq of dorsal horn tissue from mice that had undergone spinal nerve ligation revealed an increased expression of the *Glra2* gene after 7 days (Yu et al., 2019). Altogether, these results suggest that the spinal inhibitory dysfunction after peripheral nerve injury may involve changes in α2GlyRs expression.

### Glycine Receptors Composed of α3 Subunit

Increasing evidence points to a critical role of dorsal horn α3-containing GlyRs in chronic inflammatory pain (Zeilhofer et al., 2021; Figure 1C). Initial electrophysiological studies showed that the activation of EP2 receptors (EP2-R) by prostaglandin E2 (PGE_2_) decreased Gly-IPSCs in dorsal horn neurons through a PKA-dependent mechanism (Ahmadi et al., 2002). Using mice lacking the expression of α3GlyRs (α3^–/–^) or EP2-R (EP2^–/–^), two follow up studies demonstrated a direct involvement of α3GlyR and EP2-R in glycinergic dis-inhibition in inflammatory pain (Harvey et al., 2004; Reinold et al., 2005). Electrophysiological recordings showed that the Gly-IPSC inhibition driven by EP2-R activation described in wild-type mice was absent on tissue from α3^–/–^ or EP2^–/–^ mice. Moreover, behavioral studies showed that both α3^–/–^ or EP2^–/–^ animals developed a similar reduced time window of pain hypersensitivity induced by peripheral inflammation. Moreover, both α3^–/–^ or EP2^–/–^ mice were not significantly sensitized by intrathecal application of PGE_2_. Interestingly, α3^–/–^ mice showed a normal acute nociception (Harvey et al., 2004; Acuña et al., 2016) and displayed an unaltered pain sensitization after peripheral nerve injury (Hösl et al., 2006).

Using recombinant expression of mutant GlyRs, Harvey et al. (2004) identified the S346 residue within the ICD of α3GlyRs as the residue targeted by PKA. The recent generation of a knock-in mouse line carrying the S346A mutation in the *Glra3* gene allowed direct examination of the relevance of this chronic pain mechanism *in vivo* (Werynska et al., 2021). Behavioral experiments demonstrated that α3GlyR-S346A mice are devoid of pain sensitization elicited by intrathecal PGE_2_ and display a diminished inflammatory pain hyperalgesia. The *in vivo* observations correlated well with electrophysiological recordings performed on dorsal horn tissue from α3GlyR-S346A mice, which did not show the PGE_2_-mediated reduction of Gly-IPSCs seen in wild-type mice. These results confirm the role of PKA-dependent phosphorylation of α3GlyRs in inflammatory pain and highlight the significance of α3-containing GlyRs as pharmacological targets (Cioffi, 2017; Zeilhofer et al., 2018, 2021).

The relevance of α3GlyRs has fostered studies exploring the molecular mechanisms connecting the S346 phosphorylation to decreased glycinergic currents (Figure 1E). Using voltage clamp fluorometry, Han et al. (2013) proposed that the S346 phosphorylation promotes a wave of conformational changes that affect the glycine-binding site. Further studies revealed a direct link between S346 phosphorylation and alterations of ion channel conductance (Moraga-Cid et al., 2020). Single-channel experiments showed that the optogenetic or chemical activation of PKA signaling reduced the unitary conductance of wild-type α3GlyRs. The lower conductance was mirrored by a phospho-mimetic α3GlyR construct (S346E) and was reproducible on chimeric channels lacking the α3GlyR ECD. The single channel results indicated a reduction of the unitary current amplitudes by about 35% in wild-type or phospho-mimetic receptors, which is similar to the decrease of the Gly-IPSCs observed in dorsal horn slices (Ahmadi et al., 2002; Harvey et al., 2004; Acuña et al., 2016). Besides direct alterations on the ion channel function, phosphorylation processes have also been associated with changes in receptor mobility. In spinal neurons expressing GlyRs in fusion with the fluorescent protein Dendra2, Niwa et al. (2019) found a differential effect of PKA signaling on synaptic α1β or α3β GlyRs. Single-particle tracking experiments showed that forskolin treatment increased the synaptic diffusion coefficient of Dendra2-α3GlyRs, while Dendra2-α1GlyRs remained unaltered. Thus, these experiments suggest that S346 phosphorylation may also promote glycinergic dis-inhibition by increasing the mobility of synaptic α3GlyRs.

### Glycine Receptor β Subunit

To date, there is no direct evidence connecting the β GlyR subunit with sensory processing or chronic pain. However, the lack of evidence does not mean that β subunits are irrelevant in the control of pain. As the β GlyR is modified by post-translational modifications that adjust its subcellular localization (Specht et al., 2011; Grünewald et al., 2018) and function (Caraiscos et al., 2002; Muñoz et al., 2021), it is likely that pain-related plasticity phenomena associated with glycinergic neurotransmission are linked with modifications of the β subunits. Future studies and novel models (Maynard et al., 2021) may contribute to clarify the specific roles of β GlyR subunit on pain signaling.

## Conclusion

The results summarized here allow us to conclude that GlyR subtypes differentially contribute to nociception and chronic pain. The current evidence strongly suggests that post-translational modifications on synaptic α1β and α3β GlyRs are involved in processes of spinal glycinergic dis-inhibition and chronic inflammatory pain. On the other hand, the roles of the α2 and β subunits remain largely unknown. The diverse molecular mechanisms involved suggest a highly dynamic plasticity of GlyRs and of other proteins of the glycinergic system. The signaling pathways controlling GlyR phosphorylation appear to be critical for the fine tuning of the inhibitory synaptic strength. So far, the available data suggest that GlyR phosphorylation works as a molecular switch regulating plasma membrane expression, synaptic localization, and ion channel function. Whether these mechanisms act in parallel or independently at glycinergic synapses affected by pain-related signals remains to be determined.

Despite an increasing number of recent reports, it should be noted that the characterization of the GlyR subtypes roles in nociception and chronic pain is far from complete, as many open questions in the field have not yet been addressed. Of significant concern is the relevance of glycinergic tonic inhibition and presynaptic GlyRs in pain control. Whether supra-spinal GlyR subtypes (Husson et al., 2014; Muñoz et al., 2018) or GlyRs expressed in non-neuronal cells (Van den Eynden et al., 2009) also participate in the regulation of pain is another question that is almost entirely unexplored. Similarly, the expression and potential significance of GlyRs on primary afferents remains controversial (Wilke et al., 2020; Yao et al., 2021). Finally, the relevance of autoantibodies targeting GlyRs in pain control in humans is largely unknown (Crisp et al., 2019; Rauschenberger et al., 2020). The identification and characterization of novel protein kinases, protein-protein interactions and post-translational modifications involved in the regulation of GlyRs may open exciting new avenues in pain research. Hopefully, future progress in terms of the development of subunit selective GlyR pharmacological agents and the generation of novel genetic models will clarify some of these issues and expand our current knowledge in the coming years.

## Author Contributions

VSM, AS, EU, GM-C, and GEY participated in the conception of the review and wrote the manuscript. All authors contributed to the article and approved the submitted version.

## Conflict of Interest

The authors declare that the research was conducted in the absence of any commercial or financial relationships that could be construed as a potential conflict of interest.

## Publisher’s Note

All claims expressed in this article are solely those of the authors and do not necessarily represent those of their affiliated organizations, or those of the publisher, the editors and the reviewers. Any product that may be evaluated in this article, or claim that may be made by its manufacturer, is not guaranteed or endorsed by the publisher.
